# Quality of maternal obstetric and neonatal care in low-income countries: development of a composite index

**DOI:** 10.1186/s12874-019-0790-0

**Published:** 2019-07-17

**Authors:** Danielle Wilhelm, Julia Lohmann, Manuela De Allegri, Jobiba Chinkhumba, Adamson S. Muula, Stephan Brenner

**Affiliations:** 1 Heidelberg Institute of Global Health, Ruprecht-Karls Universität Heidelberg, Im Neuenheimer Feld 130.3, 69120 Heidelberg, Germany; 2 Department of Public Health, School of Public Health and Family Medicine, College of Medicine, University of Malawi, Private Bag 360, Chichiri, Blantyre 3 Malawi

**Keywords:** Composite index, Maternal health, Neonatal health, Quality of care, Low income countries

## Abstract

**Background:**

In low-income countries, studies demonstrate greater access and utilization of maternal and neonatal health services, yet mortality rates remain high with poor quality increasingly scrutinized as a potential point of failure in achieving expected goals. Comprehensive measures reflecting the multi-dimensional nature of quality of care could prove useful to quality improvement. However, existing tools often lack a systematic approach reflecting all aspects of quality considered relevant to maternal and newborn care. We aim to address this gap by illustrating the development of a composite index using a step-wise approach to evaluate the quality of maternal obstetric and neonatal healthcare in low-income countries.

**Methods:**

The following steps were employed in creating a composite index: 1) developing a theoretical framework; 2) metric selection; 3) imputation of missing data; 4) initial data analysis 5) normalization 6) weighting and aggregating; 7) uncertainty and sensitivity analysis of resulting composite score; 8) and deconstruction of the index into its components. Based on this approach, we developed a base composite index and tested alternatives by altering the decisions taken at different stages of the construction process to account for missing values, normalization, and aggregation. The resulting single composite scores representing overall maternal obstetric and neonatal healthcare quality were used to create facility rankings and further disaggregated into sub-composites of quality of care.

**Results:**

The resulting composite scores varied considerably in absolute values and ranges based on method choice. However, the respective coefficients produced by the Spearman rank correlations comparing facility rankings by method choice showed a high degree of correlation. Differences in method of aggregation had the greatest amount of variation in facility rankings compared to the base case. Z-score standardization most closely aligned with the base case, but limited comparability at disaggregated levels.

**Conclusions:**

This paper illustrates development of a composite index reflecting the multi-dimensional nature of maternal obstetric and neonatal healthcare. We employ a step-wise process applicable to a wide range of obstetric quality of care assessment programs in low-income countries which is adaptable to setting and context. In exploring alternative approaches, certain decisions influencing the interpretation of a given index are highlighted.

**Electronic supplementary material:**

The online version of this article (10.1186/s12874-019-0790-0) contains supplementary material, which is available to authorized users.

## Background

Over the last two decades, there has been significant progress in reducing the number of maternal deaths globally, with a 45% decrease in the maternal mortality ratio (MMR) from 1990 to 2013. Despite multiple interventions to improve both maternal and neonatal healthcare services in low-income countries, great disparities remain between high and low-income countries with an average lifetime maternal mortality of 1 in 38 compared to 1 in 3700 respectively [1]. The disparity persists in relation to neonatal deaths with 99% of 2 million annual neonatal deaths occurring in low and middle-income countries [2, 3]. The majority of maternal and neonatal deaths occur during the intrapartum and immediate postpartum periods with obstetric hemorrhage as the primary cause [3–5]. While studies demonstrate greater access and utilization of maternal and neonatal health services in low-income countries (LIC), mortality rates remain high with poor quality increasingly scrutinized as the potential point of failure in achieving expected goals [6–9]. Thus, the evaluation of the quality of obstetric care, especially in LIC, has garnered increasing attention [10].

Defining appropriate measurements to assess quality can be challenging due to the multi-dimensional nature of quality [11]. Although attempts have been made, there remains a lack of consensus on appropriate measurements and data sources to be used in low-income countries [6]. The vast number and complexity of existing quality indicators, while useful for monitoring specific clinical settings, can have limited utility in comprehensive monitoring due to the amount of information needed to be processed [12]. One way to simplify is through composite indicators or indices, which combine individual indicators into a single index reflecting a more complex underlying concept (e.g. quality of care) [11, 12]. Composite indices allow various perspectives to be reflected simultaneously and thus facilitate the comparison of quality performance between facilities or health systems over time [11, 13]. The resulting composite scores can be easily communicated to diverse stakeholders such as providers, health managers, or purchasers of healthcare [12].

In respect to obstetric care, individual quality indicators or simple indices are commonly used in tracking progress along specific sub-components of care. However, long lists of indicators can fall short on providing condensed information on quality that more easily facilitate comparisons across facilities, health programs, or countries [14]. As quality improvements in one specific area of care do not necessarily correlate to improvements in other areas, composite indices resulting in a single score can account for a multitude of relevant indicators across multiple quality dimensions [12, 15]. However, to ensure a composite index accurately reflects a multidimensional concept, it is prudent to adhere to a step-wise approach based on transparent methodologies and statistical consistency, allowing for replication and application across stakeholders and environments [11, 14].

There is a wide range of approaches and applications related to evaluation of obstetric and neonatal quality of care in LIC settings. In the context of quality improvement program evaluations, process and input indicators are commonly reported individually, especially if sub-components of obstetric care are being assessed [13, 16, 17]. Otherwise, these individual indicators are summarized into simple indices each representing a sub-component of obstetric care (e.g. infection prevention, third stage labor management, respectful care, etc.) [18–20]. A few evaluation studies apply more complex statistical approaches, such as standardization of scores or principal component analysis, in generating quality indices but are limited in scope [21, 22]. In contexts where quality of care indicators are used as an integral part of the intervention, such as in performance-based financing, indicator weights are common means to reflect the relative contributions of single quality aspects in the resulting quality score [23, 24]. Given the complexity of obstetric care, evaluations would need to rely on a range of data sources to accurately reflect the multi-dimensionality of quality of care. Some standardized approaches (e.g. Bologna score) collect data with only patient exit interviews or other single data sources, which cannot capture all relevant dimensions of quality required for a robust comprehensive evaluation [25, 26]. In recent program evaluations [19, 27], quality of care has been assessed in a more multifaceted way using facility inventories, interviews, and structured observation checklists, but a standard approach in combining the indicators into one meaningful composite index has not yet emerged.

There appears to be a lack of composite indices of obstetric quality of care that can be easily applied to LICs demonstrating a multidimensional concept while following a transparent process. To address this gap, we employ an existing conceptual framework reflecting the measurement of quality of care in order to develop an index resulting in composite scores, which can then be used to compare obstetric and neonatal quality of care among facilities. This article attempts to illustrate the step-wise development of a composite index based on current standards of construction with the goal to produce a single score reflecting the multidimensional aspect of maternal obstetric and neonatal quality of care. Using a systematic approach starting from a set of quality of care indicators to form different composite indices, we further demonstrate how various methodological approaches affect the resulting score.

## Methods

### Data sources

To illustrate the development of this obstetric quality of care score, we used data taken from the baseline assessment of the Results Based Financing for Maternal and Newborn Health (RBF4MNH) program in Malawi [28]. This evaluation included a sample of 33 Emergency Obstetric Care (EmOC) facilities (five hospitals, 18 health centers) offering obstetric and newborn care services located in four districts: Balaka, Dedza, Mchinji, Ntcheu. Baseline data was collected in 2013 prior to the start of the implementation of RBF4MNH and included four different data collection tools: a facility inventory, structured patient-provider observations, a structured interview with health workers, and a structured exit interview with women who recently delivered at the facility. All data was collected by trained research assistants. The facility inventory assessed the availability of equipment, essential medications, guidelines, emergency transportation and human resources. The provider-patient sample consisted of a total of 82 direct observations of uncomplicated delivery cases and assessed birth attendants’ adherence to clinical guidelines during routine obstetric care. Interviews were conducted with a total of 81 midwives and midwifery nurses, assessing health worker satisfaction in the work place and their experiences with supervision and training. The exit interview sample consisted of 204 women who delivered at these facilities; interviews assessed women’s experience receiving obstetric care at the facility and their perceptions of the quality of care received.

### Composite index development approach

We employed the step-wise approach outlined by the Organization of Economic Cooperation and Development (OECD) guidelines for composite index development [12]. Although developed for high-income countries, the identified standards are fully applicable to the context of LICs. The OECD guidelines includes the following steps with slight modifications: 1) developing a theoretical framework; 2) metric selection; 3) imputation of missing data; 4) initial data analysis 5) normalization 6) weighting and aggregating of selected variables; 7) uncertainty and sensitivity analysis of resulting composite score; 8) and deconstruction of score into its components [12, 14].

Based on this approach, we developed a base composite index resulting in a composite score for each facility and tested alternatives by altering the decisions taken at different stages of the construction process [12, 14]. Table 1 provides an overview of different approaches at each step to further illustrate the base and alternative index scenarios taken to formulate the composite scores.

**Table 1:** Steps in developing base case composite indicator with alternative methods

STEPS		
1. Theoretical Framework: Quality Matrix		
2. Metric Selection: Literature Review/Expert Opinion		
3. Missing data imputation: imputation by mode for binary variables or mean for continuous variables		
4. Initial Data Analysis: Review outliers/directionality		
Indicators within cells	Base Case	Alternative
5a. Normalization:	Binary categorization of non-binary cell indicators	A. Rescaling of non-binary cell indicators (Min-max)
5b. Weighting:	Equal weighting	
5c. Aggregation:	Additive linear aggregation of indicator scores	B. Geometric aggregation of indicator scores
Cells within matrix	Base Case	Alternative
6a. Normalization:	Rescaling of cell scores (Min-max)	C. Standardization of cell scores (Z-scores)
6b. Weighting:	Equal weighting	
6c. Aggregation:	Additive linear aggregation of cell scores	D. Geometric aggregation of cell scores
7. Uncertainty/Sensitivity Analysis: comparison base case against alternative methods		
8. Deconstruction: explore individual indicators contribution to composite score		

#### Conceptual framework

The conceptual framework, which provided the basis of choosing single indicators to contribute to the composite index, was slightly modified from a multidimensional matrix measuring quality of care first introduced by Maxwell [29] and later refined by Profit et al. [14] (See Table 2). We consider this matrix ideal for the purpose of measuring quality of care as it incorporates two complementary approaches of measuring quality of care. This results in a quality matrix which sufficiently reflects the dynamic process of healthcare delivery [14, 31]. The matrix includes the six key dimensions of quality of care as initially outlined by the Institute of Medicine (IOM) [32] and subsequently adapted by the World Health Organization (WHO): effective, efficient, accessible, acceptable/patient-centered, equitable, and safe [30]. These are complemented by the three quality of care elements first described by Donabedian: structure, process, and outcome [33]. We felt that the definition of the WHO dimensions correlated best with the contextual environment of LICs with the aspect of timeliness included under the WHO quality dimension of accessibility, which also considers that healthcare services need to occur in a setting that is equipped with adequate resources to meet the needs of the community.

**Table 2:** Conceptual Framework^a,b^

	EFFECTIVE	EFFICIENT	ACCESSIBLE/ TIMELY	PATIENT-CENTERED/ ACCEPTABLE	EQUITABLE	SAFE
	health care that is adherent to evidence based guidelines resulting in improved health outcomes	delivering health care which maximizes resource use and avoids waste	health care that is timely, geographically reasonable, with appropriate skills and resources	health care that takes into account preferences of service users and community culture	health care quality does not because of personal characteristics such as gender, race, or socioeconomic status;	delivering health care which minimizes risks and harm to service users.
STRUCTURE						
PROCESS						
OUTCOME						

^a^ Adapted from Profit J, Typpo KV, et al. Improving benchmarking by using an explicit framework for the development of composite indicators: an example using pediatric quality of care. Implement Sci. 2010;5 (1):13 [14]

^b^ WHO, editor. Quality of care: a process for making strategic choices in health systems. Geneva: WHO; 2006. 38 p [30]

#### Metric selection

Guided by this conceptual framework, the indicator selection process was based on a literature review focused on obstetric and neonatal care quality indicators. The starting point was the recent WHO publication on “Standards for Improving Quality of Maternal and Newborn Care in Health Facilities” [34] with a set of quality of care indicators identified through literature review, expert consultations, and a consensus-building Delphi process representing 116 maternal health experts in 46 countries. We further examined additional sources of maternal and neonatal quality of care indicators [4, 35–45] to identify any further indicators that had not been specified in the WHO document. Using multiple sources in combination with the WHO document, we identified an initial set of indicators most relevant to obstetric and neonatal care quality. Starting with this indicator selection, the content and definition of each indicator was reviewed with duplicated indicators removed or redundant indicators combined (e.g. adequate supervision available vs. number of supervisory visits).

We mapped the resulting indicators by assigning them to the cells provided by the conceptual quality of care matrix (Table 2). Generally, there was little to no overlap in assigning indicators to single matrix cells. In situations where an indicator could be assigned to more than one cell, consensus between co-authors was sought for the most appropriate indicator assignment given both the dimension definition and content suggested by the reviewed literature. For example, “availability of clean water” could conceptually fall under “accessible” to represent access to water or “safe” to highlight the importance of clean water. Ultimately, the indicator was assigned to the safe dimension to represent “sanitation and hygiene”. For the following steps we transition from the literature to the existing data from Malawi as described above.

#### Imputation of missing data

Generally, data quality in terms of completeness was high. Most missing values were due to certain data collection tools not being applied at certain facilities. As our aim was to develop a composite score including information from each of the different data sources, we included only facilities where all four data collection tools were actually applied resulting in a final sample of 26 facilities out of a total 33 EmOC facilities. The vast majority of missing values occurred in variables stemming from direct observations where observers were asked to enter “1” if they observed a certain task and “0” if they did not, in the course of the observation. Supervision and debriefings during data collection revealed that the latter tended to be an issue, with observers not being aware of the implications of not entering zeros for non-observed behavior at the end of the observation. We are, therefore, highly confident that missing values on these variables actually reflect non-observation of behavior and replaced missing values with “0” accordingly. We are further confident that the small remaining number of missing values can be assumed to be missing at random and were replaced with the respective sample mode (or sample mean for the one continuous variable with missing values). Due to the nature of how the data was collected and the missing values, multiple imputation would not have been appropriate as an alternative [46]. Therefore, we searched for a proxy variable that would be a close substitute for the missing data in the original variable [47]. When it was not possible to identify an appropriate proxy variable, we used the mode for binary variables as an alternative for missing data in the direct observations. In addition, we provided a second alternative by coding the missing values in the direct observations as “1” thus providing a full range of possible outcomes (detailed information on missing data and results of using alternative missing imputation methods is provided in the Additional file 1).

#### Initial data analysis

As the composite index was intended to be calculated at facility level, we aggregated the data from individual- level data collection tools which measured information at the individual to the facility level. We did this by averaging data across all individual-level observations (i.e. cases, interviews) for each variable and facility. This resulted in scores between 0 and 1. For reasons of simplicity, these proportions were then retransformed into binary variables using a 0.5 cut-off (i.e. “0” for less 0.5, “1” for 0.5 or greater). The few continuous variables were averaged. This resulted in one observation for each variable and each facility, which was necessary to combine the data sets.

In the following step, we matched the variables contained in the available datasets with the mapped matrix indicators. Once matched, we analyzed the variables contained in each matrix cell for internal consistency by correlating each variable pair within the cells. Variables of a given cell with correlations > 0.7 were re-evaluated and were merged into one single variable, in cases where the variables measured approximately the same quality construct and were consistent with the conceptual framework.

#### Indicators within cells: normalization, weighting and aggregation

Due to the necessity of a uniform scale for aggregation, normalization of the indicator values is required when different units of scale exist [12, 14]. As the vast majority of variables was binary, in the base case, we transformed the couple remaining non-binary variables using cut-off values supported by standards reported in the literature. To define the number of skilled birth attendants per facility, a cut-off value of at least 3 was used based on the literature and requirements of the program [48]. For the other continuous variable, time from arrival to contact with the provider, we used the median time of 20 min as our cut-off value. The remaining variables were ordinal variables with the median used as a cut-off value. For Alternative A (Table 1), we rescaled the few non-binary variables to a range of values between 0 and 1 (see below).

To identify weights, we considered data-driven methods (e.g. principal component analysis) relatively inappropriate given our variables mainly represented measures of adherence to universally established quality of care standards [12]. Thus, statistically derived weights may have assigned more importance to readily measurable or easily achievable input or process measures relevant to the observed context, but independent of the defined standards, making comparability across settings difficult. Therefore, we identified weights using expert ratings identified by the WHO Delphi study [34]. However, as these indicator ratings varied only minimally and thus did not sufficiently support a clear weighting pattern for indicators identified by the matrix, we applied equal weights. Additional publications on quality of care indicator weights almost uniformly suggested the use of equal weights. [12, 49, 50].

For the base case scenario, the indicators within each matrix cell were then aggregated using an additive approach, meaning that the values for each indicator within a cell were added together to reflect a raw sum with the maximum sum (i.e. cell score) varying between cells depending on the total number of indicators within a given cell. In the respective Alternative B (Table 1), we used geometric instead of additive aggregation (see below).

#### Cells within matrix: normalization, weighting, and aggregation

In a next step, we further combined the cell scores into a single composite score. As the maximum cell scores (ranging from 6 to 19) differed depending on the number of indicators identified for each cell, we rescaled each cell score based a range from 0 to 1, except in Alternative C where Z-score standardization was used to rescale [12]. Rescaling the cell scores ensured each cell contributes equally to the overall composite score. These rescaled scores were subsequently aggregated, using equal weights to obtain an overall composite score ranging from zero to twelve. In the respective Alternative D (Table 1), we replaced the additive aggregation of cell scores with geometric aggregation (see below).

#### Uncertainty and sensitivity analysis

A number of uncertainties based on decisions, such as normalization and aggregation methods, taken at various steps can influence the outcome of a composite score. Therefore, we calculated the outcomes with theoretically equally valid but different decisions to evaluate for a practically relevant difference [51]. Given these many steps and decisions taken in response to the underlying data, we further explored possible uncertainties introduced by not opting for an alternative approach at a given step [51]. Therefore, we created a set of alternative composite indices that differed in one decision step and compared these to our base composite index. The four alternative approaches are as follows (see also Table 1):

##### Alternative A

Instead of transforming non-binary variables (ordinal or continuous) into a binary form, we re-scaled them to a range between 0 and 1. This alternative approach could increase distortion by extreme values, but at the same time widens the contribution of variables that have a narrow range of values across the sample, thus better reflecting the actually measured information and underlying variance of these variables [12].

##### Alternative B

Geometric aggregation (i.e. multiplying indicator values) of indicators to obtain a cell score, rather than arithmetic aggregation. With this alternative, “0” values in single indicators can no longer be compensated by the remaining indicators, which would have a larger effect on the outcome in the case of binary measurements.

##### Alternative C

Standardization using Z-scores in each cell to achieve normalization, which converted cell score values to a normally distributed scale with a mean of 0 and a standard deviation of 1. Standardization of cell indicators with extreme values will have a greater effect in the resulting composite score [12].

##### Alternative D

Geometric aggregation to combine cell score into a composite score, decreasing the extent of compensation of low cell score values by high values.

Our sensitivity analysis consisted of a descriptive comparison of the scores and applying ranks to each studied facility using the base and alternative scores. Robustness of facility ranking using the base index compared to the alternative indices was determined using Spearman rank correlation.

#### Deconstruction

We deconstructed the base and alternative composite scores by evaluating each cell within the matrix, comparing sample means and confidence intervals (95% confidence intervals, +/− 2 standard deviations) using base and alternative scores. Furthermore, we applied the same methods to evaluate the elements of structure, process, and outcome.

## Results

We begin with presenting the results of the literature review exercise in choosing indicators, followed by the empirical results of the data analysis.

### Indicator selection

From the reviewed literature, we initially identified 271 possible indicators representing the quality of obstetric and neonatal care (Additional file 2). Mapping across our conceptual matrix, at least one identified indicator covered each of the quality of care elements and dimensions. When matching our available data from Malawi to this literature-based comprehensive indicator set, we failed to sufficiently match two of the six dimensions: efficiency and equity. We considered possible indicators for efficiency and equity using our data, but insufficient numbers of variables in our dataset reflecting efficiency and equity would not properly represent these dimensions in comparison to other dimensions. Our final result yielded 85 indicators distributed among 4 quality dimensions representing structure, process, and outcome elements with the data available to us (Additional file 3).

### Uncertainty and sensitivity analysis

**Fig. 1:**
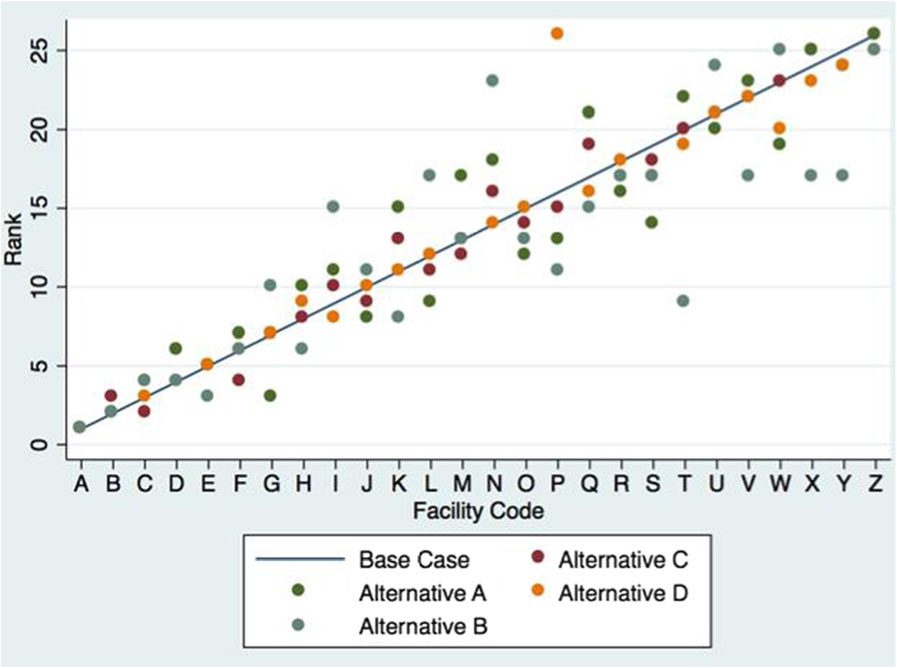
Facility Rankings

Table 3 presents the base and four alternative composite scores for each of the 26 facilities (labeled A through Z in descending order per rank of facility-specific base score). Correspondingly, the ranks of the base (line) and alternative scores (dots) are presented in Fig. 1 Facility Rankings. The scores and ranks vary considerably by method choice and noted differences are as follows:

- **Base vs. alternative A:** Rescaled ordinal or continuous variables are compared to binary variables (base case). Alternative A scores are slightly more condensed and therefore show less variation across facilities. This method reduces the extreme ends of the scale and extremely well performing facilities are no longer seen as potential outliers. Despite the narrow distribution of values in the underlying data, the variation in values remains minimal. This method showed no significant outliers in facility rankings compared to the base case (Fig. 1).
- **Base vs. alternative B:** Additive aggregation (base case) is compared to geometric aggregation of binary indicators into cell scores. With the same maximum possible points as the base case, in the absence of perfect quality, geometric aggregation leads to substantially lower scores in each cell than additive aggregation, and, therefore to lower total scores. In addition, this method created outliers in facility rankings compared to the base case. This can be seen best with Facility N (Fig. 1), which ranked 14th in the base case, but dropped to 23rd due to not meeting all indicators in 9 of the 12 cells. Although this facility obtained some or most of the indicators in each cell, it did not obtain all indicators in the majority of cells, which caused this facility to be most affected by this method.
- **Base vs. alternative C:** Rescaled cell scores using the Min-max method (base case) is compared to standardization using Z-scores for each cell, which expands the underlying scale to a normal distribution curve. Although this allows for easier identification of exceptionally good and poor performing facilities, it is a relative metric, only allowing for comparison of facilities against each other, but does not give the user a way to easily identify how well the facilities are performing in absolute terms (e.g. against some standard, against another sample). This method also shows no significant outliers in facility rankings compared to the base case.
- **Base vs. alternative D:** Additive aggregation of the cell scores (base case) is compared to geometric aggregation into a composite score. The maximum scale range is now 0–1 with almost all facilities identified as poor performers. This method is extremely sensitive to a low performance in a given cell score and therefore does not allow for much differentiation between facilities. This method also showed a significant difference in facility rankings compared to the base case most notable for facility P, which was ranked 16th in the base case, but drops to 26th when using the geometric aggregation at the cell level. This facility scored “0” in the patient-centered structure cell, which then resulted in a total score of “0” using this method.

**Table 3:** Base and Alternative Composite Scores by Facility

Facility	Base Case	A. Min-max Normalization (indicators)	B. Geometric Aggregation (indicators)	C. Z-score Standardization (cells)	D. Geometric Aggregation (cells)
Scale Range	0–12	0–12	0–12	not applicable	0–1
A	11.07	9.81	2.37	11.20	0.33
B	10.67	9.59	2.13	7.97	0.22
C	10.50	9.36	1.92	8.27	0.13
D	10.03	8.89	1.92	8.27	0.13
E	9.88	8.96	2.00	5.13	0.07
F	9.83	8.75	1.79	5.39	0.06
G	9.68	9.43	1.46	4.49	0.05
H	9.37	8.28	1.79	4.10	0.02
I	9.36	8.15	1.13	2.90	0.04
J	9.21	8.74	1.29	3.75	0.02
K	9.14	7.55	1.67	1.50	0.02
L	8.99	8.43	1.00	1.99	0.02
M	8.84	7.51	1.17	1.64	0.01
N	8.50	7.50	0.79	−2.35	0.01
O	8.48	7.51	1.17	−1.46	0.01
P	8.28	7.83	1.29	−1.72	0.00
Q	8.24	7.37	1.13	−2.77	0.01
R	8.12	7.52	1.00	−2.43	0.00
S	8.10	7.63	1.00	−2.76	0.00
T	7.85	7.16	1.50	−3.80	0.00
U	7.52	7.37	0.70	−4.79	0.00
V	7.44	6.79	1.00	−5.77	0.00
W	7.39	7.39	0.50	−7.96	0.00
X	7.16	6.68	1.00	−9.16	0.00
Y	7.11	6.70	1.00	−8.40	0.00
Z	6.45	5.67	0.50	−9.99	0.00

The respective coefficients produced by the Spearman rank correlations comparing the base to each alternative score ranged from 0.90–0.99 (Table 4), indicating that there was only a small impact of aggregation and transformation decisions on the resulting facility ranking. As expected of all alternative cases, geometric aggregation at the indicator level led to the biggest discrepancies from the base case. The Z-score standardization resulted in the most similar rankings to the base case.

**Table 4:** Spearman Rank Correlations

	Base Case
A. Min-Max Normalization (indicators)	.94
B. Geometric Aggregation (indicators)	.90
C. Z-score Normalization (cells)	.99
D. Geometric Aggregation (cells)	.96

Lastly, we deconstructed the matrix to take a closer look at how cell scores differed by which method was used (Table 5). With the base score, there is a greater range in the confidence interval of the outcome element, which contains fewer indicators within each cell. Alternative A is similar to the base composite index with slightly lower score values. Alternative B shows significantly lower scores and a greater range of confidence intervals. Of particular note, is the “effective” dimension along the “process” element, with no facility able to meet every indicator within that cell as seen in Alternative B. This is also demonstrated in the dimension “accessible” along the structure element, which is the best performing dimension within the “structure” element in the base case. However, “accessible” becomes the worst performing dimension in Alternative B as the majority of facilities could meet at least some indicators, but very few could meet every indicator within this cell.

**Table 5:** Cell scores by Base and Alternative Indices

	Base Case Mean (CI)	Alternative A Mean (CI)	Alternative B Mean (CI)	Alternative C Mean	Alternative D Mean (CI)
Structure					
Effective	.80 (.73–.87)	.80 (.75–.85)	.27 (.09–.45)	0	.80 (.73–.87)
Accessible	.80 (.75–.84)	.73 (.69–.78)	.04 (.04–.12)	0	.80 (.75–.84)
Patient-centered	.60 (.48–.70)	.59 (.48–.70)	.12 (.02–.25)	0	.60 (.48–.70)
Safe	.75 (.67–83)	.70 (.62–.78)	.19 (.03–.35)	0	.75 (.67–83)
Process					
Effective	.55 (.48–.62)	.51 (.45–.57)	0	0	.55 (.48–.62)
Accessible	.82 (.75–.89)	.70 (.64–.75)	.38 (.18–.59)	0	.82 (.75–.89)
Patient-centered	.62 (.53–.70)	.44 (.38–.52)	.04 (.04–.12)	0	.62 (.53–.70)
Safe	.68 (.58–.77)	.63 (.54–.73)	.15 (.06–.30)	0	.68 (.58–.77)
Outcome					
Effective	.94 (.88–1.00)	.84 (.80–.89)	.88 (.75–1.02)	0	.94 (.88–1.00)
Accessible	.54 (.44–.64)	.46 (.39–.54)	.15 (.01–.30)	0	.54 (.44–.64)
Patient-centered	.90 (.82–.99)	.88 (.81–.95)	.81 (.65–.97)	0	.90 (.82–.99)
Safe	.75 (.65–.85)	.68 (.60–.76)	.50 (.29–.71)	0	.75 (.65–.85)
Total Scores					
Composite Total	8.74 (8.25–9.23) (range 0–12)	7.97 (7.56–8.38) (range 0–12)	3.54 (2.84–4.24) (range 0–12)	0	.043 (0.01–0.07) (range 0–1)
Structure Total	2.94 (2.73–3.14) (range 0–4)	2.82 (2.63–3.02) (range 0–4)	.62 (.31–.92) (range 0–4)	0	2.94 (2.73–3.14) (range 0–4)
Process Total	2.67 (2.42–2.91) (range 0–4)	2.28 (2.05–2.51) (range 0–4)	.58 (.27–.88) (range 0–4)	0	2.67 (2.42–2.91) (range 0–4)
Outcome Total	3.14 (2.94–3.33) (range 0–4)	2.97 (2.72–3.01) (range 0–4)	2.35 (1.99–2.71) (range 0–4)	0	3.14 (2.94–3.33) (range 0–4)

## Discussion

With this study, we present an approach towards developing a composite index for maternal obstetric and neonatal quality of care tailored to a low-income country context. Starting from an established conceptual framework, we illustrate a sequence of steps towards a maternal obstetric and neonatal quality of care composite index using literature and an existing data set. To highlight the transparency in our approach, we compare alternative scores representing different decision pathways. We believe this illustration provides a useful outline to be applied and adapted as necessary to other quality of care data sets.

### Composite score development

#### Quality of care framework

Identifying the most adequate conceptual framework is critical to creating a theoretical foundation for the assessment of complex, multidimensional constructs. Ideally, this framework should be defined a priori and guide the selection of indicators, identification of appropriate data sources, and the design of evaluation tools [12]. In this case, we were unable to fully match all matrix dimensions with the data available to us. We evaluated the possibility of linking to other data sets by reviewing the Health Management Information Systems (HMIS) data and Service Provision Assessment (SPA) obtained by the Malawi Ministry of Health [52]. In addition, we examined the Demographic Health Survey data obtained by the National Statistics Office [53, 54]. Unfortunately, the data from these surveys did not cover the specific time period when our data was collected, nor was it disaggregated by facility in order to be incorporated into our data.

The resulting composite index was limited to those aspects of quality initially captured by the data and, thus, omitting measures of equity and efficiency. Although efficiency and equality are considered essential components in improving maternal health in low-income countries, these measures are often not considered in regards to specifically the quality of maternal and neonatal health care services and rarely included in quality assessment tools as can be seen by the lack of indicators in these dimensions in our initial indicator table (Additional file 1) [45, 55, 56]. Despite the lack of attention for equity and efficiency dimensions, these aspects are important to policy makers and donors who want to ensure financial assistance is provided in an effective manner while aligning their goals with the providers of care [57, 58]. Further research could better identify appropriate and useable efficiency and equity indicators specifically related to quality of care and maternal health when aiming for comprehensive evaluation.

On the other hand, composite indices should also reflect user-friendliness, applicability, and reproducibility to inform benchmarking or performance evaluation across settings [14]. To this extent, comprehensiveness should be weighed against feasibility and practicability. To ensure easy reproducibility, an ideal composite index should consist of a limited, but relevant set of key indicators. This is especially true for the assessment of obstetric care in LICs and remains an ongoing pursuit, mainly limited by the availability of reliable and routinely collected quality measures [6, 59]. Quality of care is a widely framed construct that tries to address a variety of perspectives, therefore a universally accepted quality of care composite is difficult to achieve. The underlying quality dimensions remain rather universal, but universally accepted indicators may be difficult to achieve or differ in relevance between settings. Therefore, most obstetric care quality indices are limited in comprehensiveness by the indicators available [25].

To this regard, a more feasible approach could be to embrace these limitations and promote the development of composite indices in response to a program’s particular focus of quality of care and available data, which may differ depending on location and time. While clearly limited in universality, such composite indices may still be relevant provided they are constructed following a set of standards that maintain transparency in respect to strengths and limitations. Aligned with this more feasibility-driven approach, we tried to illustrate how such standards and transparency could be applied to the development of a composite index built upon program-specific data related to obstetric and neonatal care [28].

#### Uncertainty analysis

A major pitfall in combining indicators into composite indices are the introduction of uncertainties – knowingly or unknowingly – due to decisions taking in the normalization, weighting or aggregation of indicators, which may bias the resulting score towards desired aspects of care [60]. Statistical comparison of different decisions during the development of a composite index allows understanding of these uncertainty biases and offers the opportunity to explore how these decisions may affect the outcome of composite scores and thus facility rankings. In our illustration, all scores were relatively consistent in assigning high or low ranks to a given facility (Table 4). Theoretically, none of the alternative scenarios drastically affected the relative comparison of quality of care between studied facilities. Still, given a different sample, index differences might have been more pronounced. We point out below some strengths and weakness of the following alternatives, especially in relation to the concept of quality of care and communicating this with stakeholders.

Alternative A differs from the base score to the extent that non-binary variables were re-scaled prior to aggregation into a cell score. With these variables now contributing values between 0 and 1 (instead of somewhat arbitrary cut-off values used in the base index) the scores for all but two cells now contain decimals instead of integer information while keeping the same score range for each cell as in the base score. Overall, this led to lower absolute values for the resulting composite scores as without a set cut-off, fewer facilities meet the extreme values of “1” or “0” for these respective indicators. This relative increase in variability of the cell scores also resulted in smaller confidence intervals when compared to the binary variables of the base score (Table 5), However, if an extreme value is not excluded, this method could distort the indicator when rescaled resulting in the other smaller or larger values clustered at one end of the range [12]. This alternative is beneficial in instances where indicator content is ordinal (e.g. patient satisfaction ratings) and/or continuous (e.g. number of staff available) and a common scale needs to be created. Since the vast majority of variables in our data set were binary, transforming all variables to the binary form was more feasible. Regardless, the scores and subsequent deconstruction for Alternative A can be easily read and communicated to various stakeholders.

Alternative score C differs from the base score to the extent that cell scores were standardized prior to aggregation into the overall composite. While the rescaled cell scores in the base score result in scores ranging from 0 to 1, this standardization normalized the resulting scores around a mean of 0. Using this method for indicators or cell scores prior to aggregation, prevents any potential distortions that otherwise would have occurred by differently scaled cell means. Still, as this normalization approach does not change the actual range of the individual cell score, it allows individual facilities with extreme score values (i.e. exceptionally good or bad performance for the given score) to contribute more to their overall composite score. This might be desirable if the intention of the resulting composite is to consider exceptional performance on single indicators to be preferable. In our illustration, this element of exceptionality compared to the base score was most pronounced in the relative distance between the top-ranked facility “A” vs. the next-ranking facilities. However, this approach prevents any further comparison of performance between matrix components when deconstructing the composite averaged across facilities. Given the normal distribution introduced to the sub-scores, the resulting means will always be “0”, unless additional approaches (e.g. retransformation) are taken [12].

Alternative scores B and D differ from the base score to the extent that geometric aggregation was applied when combining indicators into cell scores or cell scores into the overall composite. This approach limits the degree to which aggregated measures can compensate for each other in offsetting a low score in one area by performing better in other areas. In alternative B, the geometric aggregation of binary cell indicators results in an all-or-nothing situation within each cell, as only one indicator value of “0” reduces the aggregated cell score to “0” [14, 15]. In our illustration, the majority of facilities did not score a value of 1 for every single cell indicator within a cell, especially in respect to quality of care related to accessible/structure (availability of functional equipment, supplies, drugs), effective/process (adherence to clinical standards), and patient-centered/process aspects (see Table 5). Whereas the base score represents quality of care more along a continuum allowing for a gradual increase in scores, geometric aggregation does not allow for this flexibility and demands more perfection, which may not be as feasible in low income environments due to lack of supplies or equipment.

This effect was even more pronounced in alternative D once geometric aggregation was applied to the rescaled cell scores. While geometric aggregation in our example reduced the variability of resulting scores (alternative D), it also had strongest effects on facility ranking (alternative B). This effect on the ranking reflects how inadequate performance in one measured item is no longer compensated, thus honoring facilities whose performance is more complete across all measured indicators. This “all-or-nothing” scenario may be desirable in implementing and evaluating health financing programs (e.g. pay for performance) where, but it may develop perverse incentives if a facility believes it cannot meet every indicator in a particular area and only focus on areas where they can achieve all indicators [61, 62]. In the context of obstetric care where omission of single processes and lack of specific equipment or supplies might have severe implications on the birth outcome [37], a composite accounting for such non-compensable single omissions may be preferable.

Lastly, we addressed alternative methods for imputing missing data (Additional file 1). We had considered more complex imputation approaches, but decided against them for various reasons, most importantly one statistical and one conceptual reason. Regarding the former, methods such as random or multiple imputation require larger sample sizes to work properly and we would have, therefore, risked further biasing our study in unknown ways. Second, from a policy perspective, we wish to illustrate how a large number of variables can feasibly be combined into an overall quality score usable in monitoring and evaluation systems, for instance. In light of this, we were reluctant to use approaches which would require more in-depth statistical knowledge to replicate.

#### Policy implications

The article illustrates how a large variety of dimensions and elements of quality of care can be combined into a meaningful and easy-to-handle composite score useful in ranking facilities by their quality level, to monitor facilities’ progress in quality improvement, and to determine which specific quality areas may need more attention. Our composite index was guided by the data sets we had available and the low-income context in which the data was obtained. Therefore, the indicators comprised not only of processes that are necessary for providing quality of care in any context, but also the inputs such as essential medicines and basic equipment that are often not readily available in a LIC [37]. As the indicators were obtained from the literature citing standards of maternal and neonatal quality of care, this index has the ability to be applied in multiple contexts. Yet, indicators may need adaptation, as is often required, to align with the local context [34]. We further hope that our example was instrumental in sensitizing readers to the implications of certain key decisions in the aggregation process.

## Conclusions

Identifying and addressing gaps in quality maternal and neonatal healthcare is an essential function in any health system in order to improve health outcomes. Providing condensed indicators of quality of care in the form of a composite index can be a useful adjunct but can also introduce biased information if not constructed carefully. In this paper, we outline and illustrate an approach to a composite index reflecting a multi-dimensional framework of maternal obstetric and neonatal healthcare. In so doing, we provide a step-wise process applicable to a wide range of obstetric quality of care assessment programs in LICs as it can be easily adapted and implemented in a given setting or context. A comprehensive matrix combining both elements and dimensions of care allows deconstruction of the composite into cell scores representing specific aspects of quality. In reflecting and exploring alternative approaches, we attempted to highlight how certain decisions influence the practicability or usefulness of a given index. By integrating known quality frameworks, we are able to develop a composite index which can communicate a multidimensional quality assessment of obstetric and neonatal healthcare to multiple stakeholders potentially informing policy changes and new interventions.

## Additional files

Additional file 1: Missing Data. The first table identifies the number and percentage of missing data followed by the base case method for imputing missing data compared to an alternative using proxy variables, which are listed if used for imputation of missing data. An additional alternative method for missing data was examined, which coded direct observation missing values as task performed (1) versus task not performed (0) in the base case. These alternative methods for imputing missing data were compared with the results, which is demonstrated in the tables containing the facility composite scores and Spearman rank correlations. (DOCX 26 kb)

Additional file 2: Initial indicator Table. This table identifies the 271 possible indicators representing the quality of obstetric and neonatal care obtained from the literature. (DOCX 27 kb)

Additional file 3: Composite Indicators. The table provides an overview of all indicators included in the composite index, which are further grouped by topic to provide a format in which specific areas of quality can easily be discerned. (DOCX 22 kb)

## Data Availability

The datasets used and analyzed during the current study are available from the corresponding author upon request.

## Abbreviations

EmOC: Emergency Obstetric Care
HMIS: Health Management Information Systems
IOM: Institute of Medicine
LIC: Low Income Country
MMR: Maternal Mortality Ratio
OECD: Organization of Economic Cooperation and Development
RBF4MNH: Results Based Financing for Maternal and Neonatal Health
SPA: Service Provision Assessment
WHO: World Health Organization

## References

[1] WHO Trends in (2014). Maternal mortality: 1990 to 2013 - estimates by WHO, UNICEF, UNFPA, the World Bank and the United Nations population division.

[2] Lawn JE, Lee ACC, Kinney M, Sibley L, Carlo WA, Paul VK (2009). Two million intrapartum-related stillbirths and neonatal deaths: where, why, and what can be done?. Int J Gynaecol Obstet.

[3] Wall SN, Lee ACC, Carlo W, Goldenberg R, Niermeyer S, Darmstadt GL (2010). Reducing intrapartum-related neonatal deaths in low- and middle-income countries-what works?. Semin Perinatol.

[4] Tripathi V, Stanton C, Strobino D, Bartlett L (2015). Development and validation of an index to measure the quality of facility-based labor and delivery care processes in sub-Saharan Africa. PLoS One.

[5] Ronsmans C, Graham WJ (2006). Maternal mortality. Who, when, where, and why. Lancet.

[6] Akachi Y, Kruk ME (2017). Quality of care: measuring a neglected driver of improved health. Bull World Health Organ.

[7] Miller S, Cordero M, Coleman AL, Figueroa J, Brito-Anderson S, Dabagh R (2003). Quality of care in institutionalized deliveries: the paradox of the Dominican Republic. Int J Gynaecol Obstet.

[8] Randive B, Diwan V, De Costa A (2013). India’s conditional cash transfer Programme (the JSY) to promote institutional birth: is there an association between institutional birth proportion and maternal mortality?. PLoS One.

[9] Lawn JE, Kinney M, Lee AC, Chopra M, Donnay F, Paul VK (2009). Reducing intrapartum-related deaths and disability: can the health system deliver?. Int J Gynecol Obstet.

[10] Morestin F, Bicaba A, Sermé J de D, Fournier P (2010). Evaluating quality of obstetric care in low-resource settings: building on the literature to design tailor-made evaluation instruments--an illustration in Burkina Faso. BMC Health Serv Res.

[11] National Quality Forum (NQF) (2009). Composite measure evaluation framework and National Voluntary Consensus Standards for mortality and safety— composite measures: a consensus report.

[12] European Commission (2008). Organisation for economic co-operation and development, SourceOECD (online service), editors. Handbook on constructing composite indicators: methodology and user guide.

[13] Profit J, Kowalkowski MA, Zupancic JAF, Pietz K, Richardson P, Draper D (2014). Baby-MONITOR: a composite indicator of NICU quality. Pediatrics..

[14] Profit J, Typpo KV, Hysong SJ, Woodard LD, Kallen MA, Petersen LA (2010). Improving benchmarking by using an explicit framework for the development of composite indicators: an example using pediatric quality of care. Implement Sci.

[15] Reeves D, Campbell SM, Adams J, Shekelle PG, Kontopantelis E, Roland MO (2007). Combining multiple indicators of clinical quality: an evaluation of different analytic approaches. Med Care.

[16] Rosen HE, Lynam PF, Carr C, Reis V, Ricca J, et al. Direct observation of respectful maternity care in five countries: a cross-sectional study of health facilities in East and Southern Africa. BMC Pregnancy Childbirth. 2015;15(1) Available from: http://bmcpregnancychildbirth.biomedcentral.com/articles/10.1186/s12884-015-0728-4. [cited 2018 Apr 14]. 10.1186/s12884-015-0728-4 PMC4657214 26596353

[17] Paxton A, Bailey P, Lobis S (2006). The United Nations process indicators for emergency obstetric care. Reflections based on a decade of experience. Int J Gynecol Obstet.

[18] Jhpiego. Mozambique: Final Report April 12, 2011–June 30, 2015 [Internet] [Internet]. Baltimore MD: Jhpiego, USAID; 2015. Available from: https://www.mcsprogram.org/wp-content/uploads/2016/02/MCHIP_Moz_FinalReport.pdf

[19] Kagema F, Ricca J, Rawlins B, Rosen H, Lynam P, Kidula N. Quality of Care for Prevention and Management of Common Maternal and Newborn Complications: Findings from a National Health Facility Survey in Kenya. Are Serv Provid Accord Int Stand. 2010; [cited 2015 Feb 23]; Available from: http://www.mchip.net/sites/default/files/Kenya%20QoC%20report%20final.pdf.

[20] Bonfrer I, Van de Poel E, Van Doorslaer E (2014). The effects of performance incentives on the utilization and quality of maternal and child care in Burundi. Soc Sci Med.

[21] Huillery E, Seban J. Performance-Based Financing, Motivation and Final Output in the Health Sector: Experimental Evidence from the Democratic Republic of Congo. 2014. hal-01071880. https://hal-sciencespo.archives-ouvertes.fr/hal-01071880/document. Accessed 12 Feb 2018.

[22] Basinga P, Gertler PJ, Binagwaho A, Soucat ALB, Sturdy J, Vermeersch CMJ (2011). Effect on maternal and child health services in Rwanda of payment to primary health-care providers for performance: an impact evaluation. Lancet..

[23] Fritsche GB, Soeters R, Meessen B (2014). Performance-based financing toolkit.

[24] USAID TRAction. Quality of Care in Performance-Based Incentives Programs: Democratic Republic of the Congo Case Study [Internet]. 2016. Available from: https://www.harpnet.org/resource/quality-of-care-in-performance-based-incentives-programs-drc-case-study/. Accessed 12 Feb 2018.

[25] Tripathi V (2016). A literature review of quantitative indicators to measure the quality of labor and delivery care. Int J Gynaecol Obstet.

[26] Chalmers B, Porter R (2001). Assessing effective care in normal labor: the Bologna score. Birth Berkeley Calif.

[27] Arscott-Mills S, Hobson R, Ricca J, Morgan L (2014). MCHIP technical summary: quality of care.

[28] Brenner S, Muula AS, Robyn P, Bärnighausen T, Sarker M, Mathanga DP (2014). Design of an impact evaluation using a mixed methods model – an explanatory assessment of the effects of results-based financing mechanisms on maternal healthcare services in Malawi. BMC Health Serv Res.

[29] Maxwell RJ (1992). Dimensions of quality revisited: from thought to action. Qual Health Care QHC.

[30] WHO (2006). Quality of care: a process for making strategic choices in health systems.

[31] Profit J, Zupancic JAF, Gould JB, Petersen LA (2007). Implementing pay-for-performance in the neonatal intensive care unit. Pediatrics..

[32] Institute of Medicine (U.S.) (2001). Crossing the quality chasm: a new health system for the 21st century.

[33] Donabedian A (2005). Evaluating the quality of medical care. 1966. Milbank Q.

[34] World Health Organization (2016). Standards for improving quality of maternal and newborn care in health facilities.

[35] World Health Organization (2015). WHO Safe Childbirth Checklist.

[36] WHO (2015). Service Availability and Readiness Assessment (SARA): An annual monitoring system for service delivery.

[37] Campbell OM, Graham WJ (2006). Strategies for reducing maternal mortality. Getting on with what works. Lancet.

[38] Faye A, Dumont A, Ndiaye P, Fournier P (2014). Development of an instrument to evaluate intrapartum care quality in Senegal: evaluation quality care. Int J Qual Health Care.

[39] Gabrysch S, Civitelli G, Edmond KM, Mathai M, Ali M, Bhutta ZA (2012). New signal functions to measure the ability of health facilities to provide routine and emergency newborn care. PLoS Med.

[40] World Health Organization and the Partnership for Maternal, Newborn and Child Health (2014) (2013). Consultation on improving measurement of the quality of maternal, newborn and child care in health facilities.

[41] Narayan I, Rose M, Faillace S, Sanghvi T (2004). The Components of Essential Newborn Care.

[42] WHO. Integrated Management of Pregnancy and Childbirth. Pregnancy, Childbirth, Postpartum and Newborn Care. A Guide for Essential Practice [Internet]. 2nd ed. Geneva: World Health Organization; 2006. Available from: https://www.who.int/maternal_child_adolescent/documents/imca-essential-practice-guide/en/. 26561684

[43] Hulton L, Matthews Z, Stones RW (2000). A framework for the evaluation of quality of care in maternity services.

[44] Brenner S, De Allegri M, Gabrysch S, Chinkhumba J, Sarker M, Muula AS (2015). The quality of clinical maternal and neonatal healthcare - a strategy for identifying “routine care signal functions”. PLoS One.

[45] Kruk ME, Freedman LP (2008). Assessing health system performance in developing countries: a review of the literature. Health Policy.

[46] Gelman A, Hill J (2007). Data analysis using regression and multilevel/hierarchical models.

[47] Huang R, Liang Y, Carriere KC (2005). The role of proxy information in missing data analysis. Stat Methods Med Res.

[48] Adegoke A, Utz B, Msuya SE, van den Broek N (2012). Skilled birth attendants: who is who? A descriptive study of definitions and roles from nine sub Saharan African countries. PLoS One.

[49] Couralet M, Guérin S, Le Vaillant M, Loirat P, Minvielle E (2011). Constructing a composite quality score for the care of acute myocardial infarction patients at discharge: impact on hospital ranking. Med Care.

[50] Bobko P, Roth PL, Buster MA (2007). The usefulness of unit weights in creating composite scores: a literature review, application to content validity, and meta-analysis. Organ Res Methods.

[51] Saisana M, Saltelli A, Tarantola S (2005). Uncertainty and sensitivity analysis techniques as tools for the quality assessment of composite indicators. J R Stat Soc Ser A Stat Soc.

[52] Ministry of Health Malawi, ICF International (2014). Malawi Service Provision Assessment (MSPA) 2013-14.

[53] National Statistical Office (Malawi) (2011). Malawi Demographic and Health Survey 2010.

[54] National Statistical Office Malawi. Malawi Demographic and Health Survey 2015-16: Key Indicators Report [Internet]. Zomba, Malawi and Rockville, Maryland, USA.: NSO and ICF International; 2016. Available from: https://dhsprogram.com/pubs/pdf/FR319/FR319.pdf.

[55] Borghi J, Ensor T, Somanathan A, Lissner C, Mills A (2006). Mobilising financial resources for maternal health. Lancet.

[56] Cromwell J (2011). Research Triangle Institute., editors. Pay for performance in health care: methods and approaches.

[57] Das A, Gopalan SS, Chandramohan D (2016). Effect of pay for performance to improve quality of maternal and child care in low- and middle-income countries: a systematic review. BMC Public Health.

[58] Savedoff WD (2010). Basic economics of results-based financing in health.

[59] Wyber R, Vaillancourt S, Perry W, Mannava P, Folaranmi T, Celi LA (2015). Big data in global health: improving health in low- and middle-income countries. Bull World Health Organ.

[60] Jacobs R, Goddard M, Smith PC (2005). How robust are hospital ranks based on composite performance measures?. Med Care.

[61] Hayward RA (2007). All-or-nothing treatment targets make bad performance measures. Am J Manag Care.

[62] Nolan T, Berwick DM (2006). All-or-none measurement raises the Bar on performance. JAMA..

