# Supplementary material for: Quality of maternal obstetric and neonatal care in low-income countries: development of a composite index
Source: BMC Med Res Methodol. 2019 Jul 17;19:154. doi: 10.1186/s12874-019-0790-0 (PMC6637560; doi:10.1186/s12874-019-0790-0)
Supplement: Supplementary file 1 — Missing Data. The first table identifies the number and percentage of missing data followed by the base case method for imputing missing data compared to an alternative using proxy variables, which are listed if used for imputation of missing data. An additional alternative method for missing data was examined, which coded direct observation missing values as task performed (1) versus task not performed (0) in the base case. These alternative methods for imputing missing data were compared with the results, which is demonstrated in the tables containing the facility composite scores and Spearman rank correlations. (DOCX 26 kb) [file 12874_2019_790_MOESM1_ESM.docx]

**Additional File 1. Missing Data**

**Missing Observations and Imputation Methods**

| **Variable** | **Number of Missing Observations (%)** | **Missing Imputation Method in Baseline Case** | **Missing Imputation Method/Proxy Variable** |
| --- | --- | --- | --- |
| **Structure-Effective** | | | |
| 1. Emergency Obstetric Care^a^ | 0 |  |  |
| 2. Newborn Care | 0 |  |  |
| 3. Vehicle Available | 0 |  |  |
| 4. Driver Available | 2 (7.7%) | Mode | Vehicle available |
| 5. Internal Supervisor | 2 (2.8%) | Mode | Mode |
| 6. External Supervisor | 2 (2.8%) | Mode | Mode |
| 7. EmOC training^b^ | 2 (2.8%) | Mode | Mode |
| 8. Newborn care training^b^ | 2 (2.8%) | Mode | Mode |
| **Structure-Accessible/Timely** | | | |
| 1. Clean water for patients | 0 |  |  |
| 2. Clean water for providers | 3 (11.5%) | Mode | Provider toilet available |
| 3. Magnesium injection | 0 |  |  |
| 4. Oxytocin injection | 0 |  |  |
| 5. Antibiotics for maternal sepsis | Ampicillin – 3 (11.5%)  Gentamycin – 0  Metronidazole – 2 (7.7%) | Mode | Benzylpencillin |
| 6. Antibiotics for neonatal sepsis | 0 |  |  |
| 7. Urine strips available | 4 (15.4%) | Mode | Glucometer Strips |
| 8. HIV tests available | 1 (3.8%) | Mode | Hemacue Test |
| 9. Thermometer available | 0 |  |  |
| 10. BP cuff available | 0 |  |  |
| 11. Pinard stethoscope available | 0 |  |  |
| 12. Ambu bag available | 0 |  |  |
| 13. Skilled staff assigned | 0 |  |  |
| **Structure-Patient-centered/Acceptable** | | | |
| 1. Patient toilet operational | 0 |  |  |
| **Variable** | **Number of Missing Observations (%)** | **Missing Imputation Method in Baseline Case** | **Missing Imputation Method/Proxy Variable** |
| 2. Patient soap available | 0 |  |  |
| 3. Maternal danger signs information | 0 |  |  |
| 4. Neonatal danger signs information | 0 |  |  |
| 5. Breastfeeding information | 0 |  |  |
| 6. Patient complaint form | 0 |  |  |
| **Structure-Safe** | | | |
| 1. Communication available | 0 |  |  |
| 2. Electricity available | 1 (7.7%) | Coded as 1  (hospital with known electricity) | Coded as 1  (hospital with known electricity) |
| 3. Provider toilet functional | 3 (11.5%) | Mode | Provider toilet available |
| 4. Provider soap available | 0 |  |  |
| 5. Surface disinfectant available | 0 |  |  |
| 6. Sterilization device functional | 0 |  |  |
| 7. Delivery kits available | 3 (11.5%) | Mode | Mode |
| 8. Sterile gloves available | 1 (7.7%) | Mode | Mode |
| **Process-Effective** | | | |
| 1. Screen for vaginal bleeding | 16 (19.5%) | Coded as 0^c^ | Examination for vaginal bleeding |
| 2. Screen for fever | 16 (19.5%) | Coded as 0^c^ | Temperature taken |
| 3. Screen for eclampsia | 16 (19.5%) | Coded as 0^c^ | Blood pressure taken |
| 4. Screen for HIV status | 16 (19.5%) | Coded as 0^c^ | Mode |
| 5. Initial vital signs taken | 16 (19.5%) | Coded as 0^c^ | Vital signs recorded in chart |
| 6. Check for signs of anemia | 16 (19.5%) | Coded as 0^c^ | Mode |
| 7. Check for proteinuria | 10 (12.2%) | Coded as 0^c^ | Mode |
| **Variable** | **Number of Missing Observations (%)** | **Missing Imputation Method in Baseline Case** | **Missing Imputation Method/Proxy Variable** |
| 8. Partograph Documentation^d^ | | | |
| Rupture of membranes | 24 (29.3%) | Coded as 0^c^ | Provider asks time of rupture (13)^e^/Mode |
| Fetal heart rate | 24 (29.3%) | Coded as 0^c^ | Newborn heart rate (20)/Mode |
| Uterine contractions | 24 (29.3%) | Coded as 0^c^ | Uterine tone post-partum (20)/Mode |
| Maternal pulse | 24 (29.3%) | Coded as 0^c^ | Maternal pulse post-partum (20)/Mode |
| Maternal blood pressure | 24 (29.3%) | Coded as 0^c^ | Maternal blood pressure post-partum (20)/Mode |
| Maternal temp | 24 (29.3%) | Coded as 0^c^ | Maternal temperature initially (24)/Mode |
| Cervical dilation | 24 (29.3%) | Coded as 0^c^ | Pelvic exam post-partum (20)/Mode |
| Amniotic fluid | 24 (29.3%) | Coded as 0^c^ | Pelvic exam post-partum (20)/Mode |
| Moulding | 24 (29.3%) | Coded as 0^c^ | Pelvic exam post-partum (20)/Mode |
| Descent of head/buttocks | 24 (29.3%) | Coded as 0^c^ | Pelvic exam post-partum (20)/Mode |
| 8. Oxytocin given | 6 (7.3%) | Coded as 0^c^ | Mode |
| 9. Examines placenta | 6 (7.3%) | Coded as 0^c^ | Mode |
| 10. Uterine massage | 6 (7.3%) | Coded as 0^c^ | Mode |
| 11. Dries baby gently | 7 (8.5%) | Coded as 0^c^ | Mode |
| 12. Skin-to-skin contact | 7 (8.5%) | Coded as 0^c^ | Mode |
| 13. APGAR at 1 minute | 7 (8.5%) | Coded as 0^c^ | Mode |
| 14. Maternal exam 1^st^ hour | 7 (8.5%) | Coded as 0^c^ | Mode |
| 15. Maternal vital signs 1^st^ hour | 7 (8.5%) | Coded as 0^c^ | Mode |
| 17. Newborn exam 1^st^ hour | 7 (8.5%) | Coded as 0^c^ | Mode |
| 18. Internal supervision | 2 (2.8%) | Mode | Mode |
| 19. External supervision | 2 (2.8%) | Mode | Mode |
| **Variable** | **Number of Missing Observations (%)** | **Missing Imputation Method in Baseline Case** | **Missing Imputation Method/Proxy Variable** |
| **Process-Accessible/Timely** | | | |
| 1. Skilled birth attendant present | 7 (8.5%) | Coded as 0^c^ | Mode |
| 2. Oxytocin available | 7 (8.5%) | Coded as 0^c^ | Mode |
| 3. Ambu bag prepared | 11 (13.4%) | Coded as 0^c^ | Mode |
| 4. PPH management^f^ | 0 |  |  |
| 5. Eclampsia management^g^ | 0 |  |  |
| 6. Time to be seen less than 20 minutes | 12 (14.6%) | Coded as 0^c^ | Mode |
| **Process-Patient-Centered/Acceptable** | | | |
| 1. Provider offers companion to stay | 14 (17.1%) | Coded as 0^c^ | Mode |
| 2. Patient reports provider offering guardian | 0 |  |  |
| 3. Provider explains exam | 16 (19.5%) | Coded as 0^c^ | Procedure explained for 2^nd^ stage (14)/Mode |
| 4. Prover explains delivery | 7 (8.5%) | Coded as 0^c^ | Procedure explained for exam(5)/Mode |
| 5. Patient reports provider explained procedures | 0 |  |  |
| 6. Ensures privacy during exam | 16 (19.5%) | Coded as 0^c^ | Procedure explained during 2^nd^ stage (14)/Mode |
| 7. Ensures privacy during delivery | 7 (8.5%) | Coded as 0^c^ | Procedure explained for exam(5)/Mode |
| 8. Patient reports privacy ensured | 0 |  |  |
| **Process-Safe** | | | |
| 1. Hand hygiene prior exam | 16 (19.5%) |  | Hand hygiene for delivery (14)/Mode |
| 2. Hang hygiene prior delivery | 7 (8.5%) | Coded as 0^c^ | Hand hygiene for exam (5)/Mode |
| **Variable** | **Number of Missing Observations (%)** | **Missing Imputation Method in Baseline Case** | **Missing Imputation Method/Proxy Variable** |
| 3. Sterile gloves for vaginal exam | 16 (19.5%) | Coded as 0^c^ | Sterile gloves for delivery (14)/Mode |
| 4. Disinfects perineum before exam | 16 (18.5%) | Coded as 0^c^ | Disinfects perineum post-partum (15)/Mode |
| 5. Sterile delivery pack available | 7 (8.5%) | Coded as 0^c^ | Mode |
| 6. Sterile cord clamp available | 7 (8.5%) | Coded as 0^c^ | Mode |
| 7. Used sterile gloves | 7 (8.5%) | Coded as 0^c^ | Uses sterile gloves for exam(5)/Mode |
| 8. Sterile device for clamping cord | 6 (7.3%) | Coded as 0^c^ | Mode |
| **Outcome-Effective** | | | |
| 1. Outcome without complications | 0 |  |  |
| 2. Provider masters skills necessary^h^ | 2 (2.8%) | Mode | Mode |
| **Outcome-Accessible/Timely** | | | |
| 1. Received care in 20 minutes | 0 |  |  |
| 2. Satisfied with medicine^i^ | 2 (2.8%) | Mode | Mode |
| 3. Satisfied with equipment^i^ | 2 (2.8%) | Mode | Mode |
| **Outcome-Patient-centered/Acceptable** | | | |
| 1. Patient satisfied with services^i^ | 0 |  |  |
| 2. Patient received family counseling | 0 |  |  |
| **Outcome-Safe** | | | |
| 1. Patient satisfied with labor room^j^ | 2 (1.0%) | Mode | Mode |
| 2. Patient satisfied with buildin^i^ | 0 |  |  |

^a^ All items that are not specifically noted are binary with indicator met if available

^b^ Indicator met if received training within the last year (categorized from never received training, more than 1 year ago, and less than 1 year ago)

^c^ Coded as not having been performed (0), these same indicators were also recoded as task being performed (1) for an additional missing imputation method

^d^ Indicator met if 5 or more of the following were documented on the partograph: rupture of membranes, fetal heart rate, uterine contractions, maternal pulse, maternal blood pressure, maternal temperature, cervical dilation, amniotic fluid, moulding of presentation, and descent of head/buttocks

^e^ Number of missing observations imputed by the proxy variable

^f^ Indicator met if health worker could give 4 or more correct answers for the treatment of post-partum hemorrhage: call for assistance, perform physical exam, give IV fluids, check patient’s vital signs, administer oxytocin, identify indication for oxytocin

^g^ Indicator met if health worker could give 3 or more correct answers for describing dangers signs of pre-eclampsia to a patient: vaginal bleeding, convulsions/seizures, headache, swelling of extremities and understand the indicator for giving magnesium

^h^ Indicator met if agreed or strongly agreed (versus neutral, disagreed, or strongly disagreed)

^i^ Indicator met if very or somewhat satisfied (versus neutral, somewhat unsatisfied, or unsatisfied)

^j^ Indicator met if patient reported 5 or above on a scale from 1-10

**Composite Scores by Facility for Base Case and Alternative Missing Imputation Methods**

| Facility | Base Case | Missing Imputation (Proxy Variable) | Missing Imputation (Task performed)^a^ |
| --- | --- | --- | --- |
| Scale Range | **0-12** | **0-12** | **0-12** |
| A | 11.07 | 11.07 | 11.07 |
| B | 10.67 | 10.67 | 10.67 |
| C | 10.50 | 10.50 | 10.56 |
| D | 10.03 | 10.03 | 10.08 |
| E | 9.88 | 9.88 | 9.88 |
| F | 9.83 | 9.83 | 9.83 |
| G | 9.68 | 9.70 | 9.68 |
| H | 9.37 | 9.37 | 9.37 |
| I | 9.36 | 9.36 | 9.41 |
| J | 9.21 | 9.21 | 9.34 |
| K | 9.14 | 9.98 | 10.62 |
| L | 8.99 | 8.99 | 8.99 |
| M | 8.84 | 9.02 | 9.07 |
| N | 8.50 | 8.50 | 8.55 |
| O | 8.48 | 8.36 | 8.36 |
| P | 8.28 | 8.28 | 8.28 |
| Q | 8.24 | 8.24 | 8.24 |
| R | 8.12 | 8.18 | 8.18 |
| S | 8.10 | 8.48 | 8.73 |
| T | 7.85 | 8.42 | 9.06 |
| U | 7.52 | 8.04 | 8.64 |
| V | 7.44 | 7.58 | 7.58 |
| W | 7.39 | 8.33 | 8.51 |
| X | 7.16 | 7.28 | 7.28 |
| Y | 7.11 | 7.11 | 7.16 |
| Z | 6.45 | 6.84 | 7.48 |

^a^ This method coded direct observation missing variables as “1” compared to “0” in the base case.

**Spearman Rank Correlation**

|  | Base Case |
| --- | --- |
| Missing Imputation  (Proxy Variable) | .95 |
| Missing Imputation  (Task performed) | .91 |
