# Supplementary material for: Quality of maternal obstetric and neonatal care in low-income countries: development of a composite index
Source: BMC Med Res Methodol. 2019 Jul 17;19:154. doi: 10.1186/s12874-019-0790-0 (PMC6637560; doi:10.1186/s12874-019-0790-0)
Supplement: Supplementary file 2 — Initial indicator Table. This table identifies the 271 possible indicators representing the quality of obstetric and neonatal care obtained from the literature. (DOCX 27 kb) [file 12874_2019_790_MOESM2_ESM.docx]

**Additional File 2. Initial Indicator Table**

|  | **Effective** | **Accessible**  **Timely** | **Patient-centered Acceptable** | **Safe** | **Efficient** | **Equitable** |
| --- | --- | --- | --- | --- | --- | --- |
| **Input**  **(Structure)** | **Guidelines available**  **for management of:**  *1. Intrapartum care/Emergency obstetric care (89.37)*^a,b^  *2. Newborn care (91.09)*  *3. Neonatal resuscitation – (91.86)*  4. Postnatal care (90.1)  5. Breastfeeding (83.18)  6. Pre-eclampsia (90.59)  7. Post-partum hemorrhage (90.76)  8. Prolonged labor (90.88)  9. Preterm labor (91.46)  10. Preterm newborn (91.41)  11. Maternal infections (91.75)  12. Neonatal sepsis (90.47)  13. Quality improvement policy -(88.08)  14. All guidelines/ policies are accessible (82.61)  **Referral system:**  *1. Functioning ambulance or emergency transport (90.35)*  2. List of network facilities in region – (89.20)  3. Standardized referral form – (89.13)  4. Agreements with referral centers (85.55)  5. Referral guidelines (89.94)  6. Staff knowledge of referral procedure  **Supervision**  1. Supervision in routine/emergency delivery care (78.65)  2. Supervision in newborn care (77.82)  3. Supervision in neonatal resuscitation (83.91)  4. Supervision in preterm labor (79.18)  *5. Adequate supervision available*  **Training on following topics**  *1. Emergency obstetric care (83.42)*  *2. Essential newborn care (83.94)*  *3. Neonatal resuscitations (87.06)*  4. Routine postnatal care (83.04)  5. Pre-eclampsia/ eclampsia (84.54)  6. Post-partum hemorrhage (84.25)  7. Prolonged labor (82.19)  8. Preterm labor (85.52)  9. Preterm newborn care (85.82)  10. Maternal infections (85.2)  11. Neonatal sepsis (84.29)  12. Referral protocols -(85.33)  13. Staff training general  14. Information systems  **Quality Improvement (QI):**  1. Designated QI team (85.38)  2. Monthly QI meetings (83.19)  3. Leaders trained in QI (81.07)  4. Staff able to provide feedback to management on QI -1 (80.82)  **Staff development:**  1. Policy for continuing professional development -1 (86.62)  2. Guideline for recognizing good performance (82.76) | **Facility structure:**  *1. Reliable water source (94.27)*  2. Warm room (77.9)  3. Operating room (90.13)  4. Area for newborn resuscitation (94.67)  5. Adequate number of birthing rooms (90.12)  6. Dedicated recovery room -1 (86.54)  **Essential Medicines available**:  *1. Anti-hypertensives /magnesium sulfate (91.8)*  *2. Oxytocin (89.55)*  *3. Antibiotics (92.15):*  *Ampicillin*  *Gentamycin*  *Metronidazole*  *Penicillin*  *Ceftriaxone*  Amoxicillin  Erythromycin  4. Antenatal corticosteroids (90.06)  5. Nevirapine  6. Antiretrovirals  7. Tetanus  8. Intravenous fluids  9. Blood products  10. Essential medications general  **Supplies Available:**  *1. Lab supplies for following tests (91.12):*  *urine protein*  *urine glucose*  *Human immunodeficiency virus (HIV)*  glucose  hemoglobiin  type/cross  bilirubin  blood culture  chemistry panel  syphilis  Malaria  Tuberculosis  *2. Essential equipment (90.33):*  *Thermometer (93.38)*  *Blood pressure machine/cuff*  *Fetal stethoscopes*  *Neonatal suction and mask (93.83)*  Vacuum/forceps (90.33)  Incubator/ warmers (89.14)  Food for preterm newborn (86.56)  Scale  Oxygen (89.37)  Light source  **Information systems:**  1. Birth/death registration system (89.44)  2. System for classifying diseases (ICD) (86.33)  3. Data collection forms available (90.34)  **Skilled staff:**  *1. Skilled birth attendants at all times -1 (89.5)*  *2. Skilled providers in sufficient numbers*  3. Staffing policy (89.05)  4. Staff knowledge of their responsibilities  **Timeliness:**  1. Triage and waiting time policy -1 (86.32) | **Health facility:**  *1. Patient bathroom available (88.94)*  *2. Hand hygiene available (89.53)*  3. Rooming in available – 1 (86.57)  4. A room or screens for privacy (94.8)  5. Space for companion (91.59)  **Information available:**  *1. Where and when mothers receive postnatal care (79.98)*  *2. Management of breastfeeding (74.49)*  3. Alternative feeding methods (78.97)  **Patient Feedback:**  *1. Patient complaint forms available for patient (77.22)*  2. Mechanism for collection of patient satisfaction (84.98)  **Training for staff:**  1. Interpersonal communication and counseling (85.74)  2. Obtaining informed consent (87.51)  3. Positive impact of companion (84.63)  4. Pain management (87.07)  **Supportive supervision:**  1. Interpersonal communication and counseling -1 (80.12)  **Guidelines/Policy:**  1. Interpersonal communication (84.69)  2. Privacy and confidentiality (89.27)  3. Informed consent (90.98)  4. Companion during delivery (91.25) | **Facility structure:**  *1. Reliable communication methods (87.14)*  *2. Energy infrastructure to meet electrical demands (89.41)*  3. Adequate sanitation facilities  **Infectious control management:**  *1. Toilets with hand washing stations (87.46)*  *2. Sterilizing facility and disinfectants for instruments (92.99)*  *3. Sterile cord ties/scissors (89.86)*  *4. Clean towels (84.83)*  *5. Sterile gloves (90.88)*  6. Safe handling of infectious waste (94.08)  7. Functioning incinerator (92.56)  8. Safe handling of sharps (93.36), 3  9. Personal protection (masks, gowns, eye protection)  **Staffing/training:**  1. Training in infection control (87.27)  2. Training in harmful practices for staff (83.00)  3. Trained pharmacist available (88.44)  **Guidelines/policy:**  1. Infection control guidelines (92.35)  2. Patient safety policy (88.08)  3. Guidelines for harmful practices (89.03)  **Documentation:**  1. Proportion of newborns with patient identifier -1 (89.15) | **Budget management:**  1. Dedicated budget for essential medicines (89.94)  2. Fuel management plan -(81.34)  3. Energy management plan -(77.26)  **Funding:**  1. Amount available from government and private sources | 1. Fee structures equitable and clearly displayed (89.07)  2. Distance from facility  3. Transportation costs for disadvantaged  4. Transportation time for disadvantaged |

|  | **Effective** | **Accessible** | **Patient-centered** | **Safe** | **Efficient** | **Equitable** |
| --- | --- | --- | --- | --- | --- | --- |
| **Process (Output)** | **History taking:**  *1. Asks about vaginal bleeding*  *2. Asks about headaches or blurred vision*  *3. Checks woman’s HIV status and offers test*  4. Asks about abdominal pain  **Physical exam/ delivery:**  *1. Takes vital signs (87.25)*  *2. Check conjunctiva/ palms (anemia)*  *3. Urine protein check*  *4. Documentation with partograph -1 (84.56)*  *- measures Heart rate/contractions/*  *blood pressure*  *- temperature*  *-vaginal exam*  5. Chest auscultation  6. Check urine for bacteria  7. Women with delay in 1^st^ stage who received oxytocin for augmentation (79.06)  **AMTSL**  *1. Receives oxytocin after delivery (86.18)*  *2. Assesses completeness of placenta and membranes*  *3. Uterine massage*  4. Cord traction  **Newborn care:**  *1. Dried immediately after birth (76.53)*  *2. Ensure newborn warmth*  *3.. Skin-to-skin contact for 1 hour (83.34)*  *4. Newborn checked for responsiveness (APGAR)*  5. Newborns receive 4 elements of essential newborn care (drying, skin-to-skin contact, delayed cord clamp, breastfeeding in 1 hour) (78.93)  6. Proportion of newborns fed in 1 hour (86.42)  7. Vitamin K and vaccination (91.23)  8. Full exam before discharge (83.13)  9. Hygienic cord care  10. Eye care  **Post-partum care**  *1. Mothers receive care for 24 hours after delivery (82.5)*  *2. Takes mother’s vital signs after birth*  *3. Palpates uterus 15 minutes after birth*  *4. Examine newborn 2 hours after birth (vitals and danger signs) (82.41)*  5. Problems documented (vital signs, breastfeeding) (84.02)  **Staff development:**  *1. Number of supervisory visits in last 3 months (80.85)*  *2. Proportion of staff assessed in last 12 months (77.54)*  *3. Regular supportive supervision and mentoring (79.24)*  4. Proportion of skilled staff who received a written job description (85.81)  5. Number of interactions per month with mentors (75.17)  6. Training in leadership and management skills (80.89)  **Emergency/complicated care Management:**  1. Instrumental delivery for obstructed/ prolonged labor  2. Women with 3^rd^/4^th^ degree perineal tears receive antibiotics (83.45)  **Quality Improvement:**  1. Leaders communicate performance to facility -1 (79.75)  2. Number of monthly meetings held to discuss QI -1 (78.69) | **Trained providers available:**  *1. Posts in the health facility filled 24 hours with appropriately trained staff (80.72)*  2. Staff have been oriented to their roles and responsibilities (78.79)  3. Assistant identified if help needed during birth  4. Skilled staff who received refresher training or mentoring in <12 months (81.8)  **Delivery preparation**  *1. Prepares oxytocin*  *2. Bag and mask for neonatal resuscitation*  3. Gloves  4. Soap and water  5. Clean towel  6. Sterile blade  7. Suction device  **Emergency care (provider knowledge):**  *1. Management of PPH:*  *-Women with PIH receive anti-hypertensives (88.02)*  *- Women with PPH receive oxytocin (89.75)*  *-Women with PPH due to retain placenta receive manual removal (80.48)*  *2. Management of eclampsia:*  *- Women with eclampsia (pre) receive mag sulfate (91.37)*  3. Neonatal resuscitation with bag-and-mask (81.1)  4. Women receive antibiotics for signs of infection (83.39)  5. Newborns with signs of infection receive antibiotics (86.94)  5. Management of preterm newborns  -Preterm newborns receive corticosteroids (86.11)  -Mothers of preterm newborns receive mag sulfate (78.52)  -Preterm newborns receive kangaroo mother care for 1^st^ week (83.35)  -Preterm newborn cared for under thermo-neutral environment (79.29)  6. Detect maternal/newborn complications  - Low/high fetal heart rate  - Breech presentation  - Shoulder dystocia  - Multiple births  **Timeliness**  1. Women reporting that they received attention within the appropriate amount of time (73.08)  2. Proportion of women who received timely care at referral facility (79.57) | **Companion:**  *1. Companion present during delivery (or encouraged) (91.76)*  **Interpersonal skills:**  *1. Explains what will happen during labor to woman and support companion*  *2. Women adequately informed about decisions regarding their care (80.82)*  3. Staff demonstrate skills of listening and responding to questions (76.67)  4. Women given opportunity to discuss concerns (82.79)  5. Staff introduced themselves (73.47)  **Patient Privacy:**  *1. Proportion of women satisfied with degree of privacy (85.63)*  **Patient Comfort:**  1. uncomplicated delivery women allowed to choose position  2. Pain relief offered (73.04)  3. Allowed to room in with baby for 24 hours (78.9)  4. Supportive care during labor (mobility, eating/drinking)  **Consent:**  1. Proportion of procedures requiring consent with consent obtained (86.9)  2. Women reporting being sought for consent (84.41) | **Hygiene:**  *1. Health care staff washes hands (87.78)*  **Clean exam:**  *1. Wears sterile gloves for vaginal exam*  *2. Clean perineum prior to vaginal exam*  3. Clean towels (84.83)  4. Hands cleaned before and after exam  **Sterile Delivery preparation:**  *1. Use of sterile equipment during childbirth*  *- Sterile blade*  *- Sterile cord ties/scissors (89.86)*  2. sterilization of instruments  **Sterile Delivery:**  *1. Ensures clean delivery technique*  *2. Use of sterile gloves during delivery*  *3. Sterile cord clamp*  **Safety of patient during delivery:**  1. Companion will call for help if needed  **Biosafety management:**  1. Safe management of health care waste (82.73)  2. Staff meet biosafety standards when administering drugs (81.85) | 1. Efficient use of resources – spending breakdown  2. Costs per case treated  3. Average length of stay  4. Cost-effectiveness rations for specific services  5. Health worker attrition rates  6. Health worker morale  7. Frequency of supervision and training | 1. Percentage of women refused care due to inability to pay (76.97)  2. Distance from clinic for disadvantaged populations  3. Utilization of essential health services by disadvantaged groups  4. Efficacy and safety analyzed for disadvantaged groups  5. Perception of exclusion/inclusion from health system |

|  | **Effective** | **Accessible** | **Patient-centered** | **Safe** | **Efficient** | **Equitable** |
| --- | --- | --- | --- | --- | --- | --- |
| **Results/**  **Outcomes** | **Morbidity/**  **Mortality**  *1. Intrapartum stillbirth rate (88.42)*  *2. Proportion of neonatal deaths due to sepsis (86.96)*  *3. Proportion of neonatal morbidity due to sepsis (77.53)*  *4. Proportion of women with pre-eclampsia/ eclampsia with a stillbirth (86.55)*  *5. Proportion of newborns with birth injuries (83.75)*  **6.** Proportion of women with pre-clampsia/ eclampsia who died (91.22)  7. Proportion of women with PPH who died (92.98)  8. Proportion of vaginal deliveries receiving blood transfusion (85.58)  9. Perinatal deaths occurring after prolonged labor (85.23)  10. Proportion of all preterm newborns who had severe neonatal morbidity (81.2)  11. Proportion of LBW newborn deaths attributed to sepsis (80.3)  12. Proportion of all women who had sepsis in postpartum period (86.15)  13. Proportion of women/newborns who had nosocomial infections (78.43)  14. Proportion of women/newborns who complete their referral (80.97)  15. Perinatal/early neonatal rate (91.39)  16. Proportion of newborns with normal body temp at first exam (85.37)  17. Proportion of newborns exclusively breastfed (85.55)  18. Proportion of newborns (preterm and term) who were exposed to antenatal corticosteroids  **Staff development:**  *1.Proportion of staff with satisfactory performance appraisal*  2. Proportion of staff highly satisfied with job (76.35)  3. Proportion of staff looking for a new job (64.26)  **Referral:**  1. Proportion of newborns who died before transfer (87.74)  2. Proportion of women who died before transfer (87.04)  3. Proportion referred without proper emergency transport (75.62) | **Patient utilization/ timeliness**  *1. Proportion of all women who reported receiving immediate attention upon arrival to health facility*  2. Proportion of women who were attended by a skilled birth attendant (90.11)  **Availability of Supplies and Equipment:**  *1. Adequate essential medicines to meet need of facility*  *2. Adequate equipment in working condition to meet needs of facility*  3. Proportion of equipment with repairs completed  4. Proportion of facilities with adequate supplies to ensure proper delivery | **Patient satisfaction**  1. Proportion of women who expressed satisfaction with the health services (80.66)  2. Proportion of women who felt adequately informed about their care (83.58)  3. Proportion of women who felt their needs were taken into consideration (80.67)  4. Proportion of women who were satisfied with the health education/ information (76.25)  5. Percentage reporting allowed to deliver in position of choice  6. Women satisfied with orientation (75.19)  7. Proportion of women making complaints (74.37)  **Counseling:**  *1. Proportion of women receiving family planning counseling before discharge (84.03)*  2. Mothers receive instruction in Breastfeeding  3. Nutrition and hygiene  4. Danger signs and where to go for complications | **Infrastructure and sanitation services:**  *1. Proportion of women/ staff satisfied with water, sanitation and energy services (76.75)*  **Near misses:**  1. Proportion of women with PPH who had a maternal near miss (77.97)  2. Proportion of women with prolonged labor who experienced maternal near-misses | 1. Proportion of women who had to contribute financially to transport -1 (68.93)  2. Mortality rates per dollars invested in health care  3. Mortality rates for different financing structures | 1. Mortality rates for lowest income quintile  2. Mortality rates for disadvantaged groups  3. Proportion of government health financing that reaches the poorest quintile  4. Extent of out-of-pocket payments  5. Incidence of impoverishment as a result of health services |

^a^ Italicized indicators matched to existing data set

^b^ Numerical rankings in parentheses given by WHO Standards for Improving Quality of Maternal and Newborn Care in Health Facilities
