# Supplementary material for: Quality of maternal obstetric and neonatal care in low-income countries: development of a composite index
Source: BMC Med Res Methodol. 2019 Jul 17;19:154. doi: 10.1186/s12874-019-0790-0 (PMC6637560; doi:10.1186/s12874-019-0790-0)
Supplement: Supplementary file 3 — Composite Indicators. The table provides an overview of all indicators included in the composite index, which are further grouped by topic to provide a format in which specific areas of quality can easily be discerned. (DOCX 22 kb) [file 12874_2019_790_MOESM3_ESM.docx]

**Additional file 3: Table S3. Composite score indicators**

|  | **Effective** | **Accessible/Timely** | **Patient-centered / Acceptable** | **Safe** |
| --- | --- | --- | --- | --- |
| **Structure** | Guideline manuals available ^a^  1. Emergency obstetric care  2. Newborn care  Emergency transport  3. Vehicle available  4. Driver available  Staff supervision available  5. internal supervisor assigned to facility  6. external supervisor assigned to facility  Training received ^b^  *7. Emergency obstetric care* ^c^  *8. Emergency newborn care* | Facility structure  1. Clean water accessible to patients  2. Clean water accessible to providers  Essential medications available  3. Magnesium injection  4. Oxytocin injection  *5. Antibiotic to treat maternal sepsis:* *ampicillin, gentamycin, or metronidazole*  *6. Antibiotic to treat neonatal sepsis: benzylpenicillin, gentamycin, or ceftriaxone*  Essential equipment/supplies available  7. Urine strips  8. Rapid HIV tests  9. Thermometer  10. Blood pressure cuff  11. Pinard (fetal) stethoscope  12. Neonatal ambu bag  Skilled staff  *13. Skilled birth attendants assigned to facility (3 or more)* | Patient sanitation  1. Patient toilet operational  2. Patient soap available  Patient Information material available  3. Maternal danger signs  4. Neonatal danger signs  5. Breastfeeding  Patient Feedback  6. Patient complaint procedure available | Facility Infrastructure  *1. Any form of communication available: landline/mobile phone or radio*  2. Electricity available  Provider Sanitation and Hygiene  3. Provider toilet functional  4. Provider soap available  5. Surface disinfectant available  Sterile equipment/supplies available  6. Instrument sterilization device functional  7. Sterile delivery kits  8. Sterile gloves |
|  | **Effective** | **Accessible/Timely** | **Patient-Centered / Acceptable** | **Safe** |
| **Process** | History Taking  1. Screen for vaginal bleeding  2. Screen for fever  *3. Screen for pre-eclampsia symptoms: headache, convulsions, blood pressure* ^h^  4. Screen for HIV status  Physical Exam and Documentation  5. Initial vital signs taken  6. Check for signs of anemia  7. Check for proteinuria  *8. Partograph documentation* ^d^  AMTSL  9. Oxytocin given  10. Examines placental membrane  11. Uterine massage until contraction  Immediate Newborn Care  12. Dries baby gently  13. Skin-to-skin contact  14. APGAR at 1 minute  Post-partum Care  *15. Maternal exam during 1^st^ hour: uterine tone or vaginal bleeding*  *16. Maternal vital signs during 1st hour: pulse or blood pressure*  *17. Newborn exam during 1^st^ hour: responsiveness or temperature*  Supervision  18. Internal supervision available  19. External supervision available | Skilled staffing  1. At least one skilled birth attendant present during delivery  Delivery Preparation  2. Oxytocin injection available  3. Neonatal bag-mask (resuscitation) available  Specific provider knowledge  *4. Post-partum hemorrhage management* ^e^  *5. Eclampsia management* ^f^  Timeliness  6. Time from arrival to provider contact less than 20 minutes | Companion  1. Offers companion to stay with woman during labor  2. Patient reports provider offering companion to be at her side  Interpersonal Skills  3. Provider explains exam procedure  4. Provider explains delivery procedure  5. Patient reports provider explaining procedures  Patient Privacy  6. Ensures privacy during exam  7. Ensures privacy during delivery  *8. Patient reports provider ensured privacy: never, sometimes, or always* | Provider Hygiene  1. Hand hygiene prior to exam  2. Hand hygiene prior to delivery  Clean Exam  3. Sterile gloves used for vaginal exam  4. Disinfects perineum before exam  Sterile delivery preparation  5. Sterile delivery pack available  6. Sterile cord clamp available  Sterile Delivery  7. Uses sterile gloves before crowning of head  8. Uses sterile device for clamping cord |
|  | **Effective** | **Accessible/Timely** | **Patient-centered / Acceptable** | **Safe** |
| **Outcome** | 1. Delivery outcome without complications  *2. Provider has mastered skills necessary for the job* ^g^ | 1. Laboring patient received attention in less than 20 minutes  *2. Providers satisfied with quantity of medicine* ^h^  *3. providers satisfied with quantity/quality of equipment* ^h^ | *1. Patient reports satisfaction of services received* ^h^  2. Patient reports receiving family counseling before leaving | *1. Patient perceived labor room as clean and of satisfactory hygiene* ^i^  *2. Patient satisfied with the physical condition of building* ^h^ |

^a^ All items that are not specifically noted as having a cut-off value are recorded as indicator met if 1 or more available

^b^ Indicator met if received training within the last year (categorized from never received training, more than 1 year ago, and less than 1 year ago)

^c^ All italicized indicators were rescaled using the Min-Max method in Alternative B

^d^ Indicator met if 5 or more of the following were documented on the partograph: rupture of membranes, fetal heart rate, uterine contractions, maternal pulse, maternal blood pressure, maternal temperature, cervical dilation, amniotic fluid, moulding of presentation, and descent of head/buttocks

^e^ Indicator met if health worker could give 4 or more correct answers for the treatment of post-partum hemorrhage: call for assistance, perform physical exam, give IV fluids, check patient’s vital signs, administer oxytocin, identify indication for oxytocin

^f^ Indicator met if health worker could give 3 or more correct answers for describing dangers signs of pre-eclampsia to a patient: vaginal bleeding, convulsions/seizures, headache, swelling of extremities and understand the indicator for giving magnesium

^g^ Indicator met if agreed or strongly agreed (versus neutral, disagreed, or strongly disagreed)

^h^ Indicator met if very or somewhat satisfied (versus neutral, somewhat unsatisfied, or unsatisfied)

^i^ Indicator met if patient reported 5 or above on a scale from 1-10
